# Supplementary material for: High-throughput Genome Wide CRISPR Knock Out mechanical sort identifies genes driving metastatic cancer cell softening
Source: bioRxiv. 2026 Feb 12:2026.02.12.705447. Preprint. [Version 1] doi: 10.64898/2026.02.12.705447 (PMC12918864; doi:10.64898/2026.02.12.705447)
Supplement: Supplement 1 [file NIHPP2026.02.12.705447v1-supplement-1.pdf]

bioRxiv preprint doi: https://doi.org/10.64898/2026.02.12.705447; this version posted February 12, 2026. The copyright holder for this preprint (which was not certified by peer review) is the author/funder, who has granted bioRxiv a license to display the preprint in perpetuity. It is made available under aCC-BY 4.0 International license.

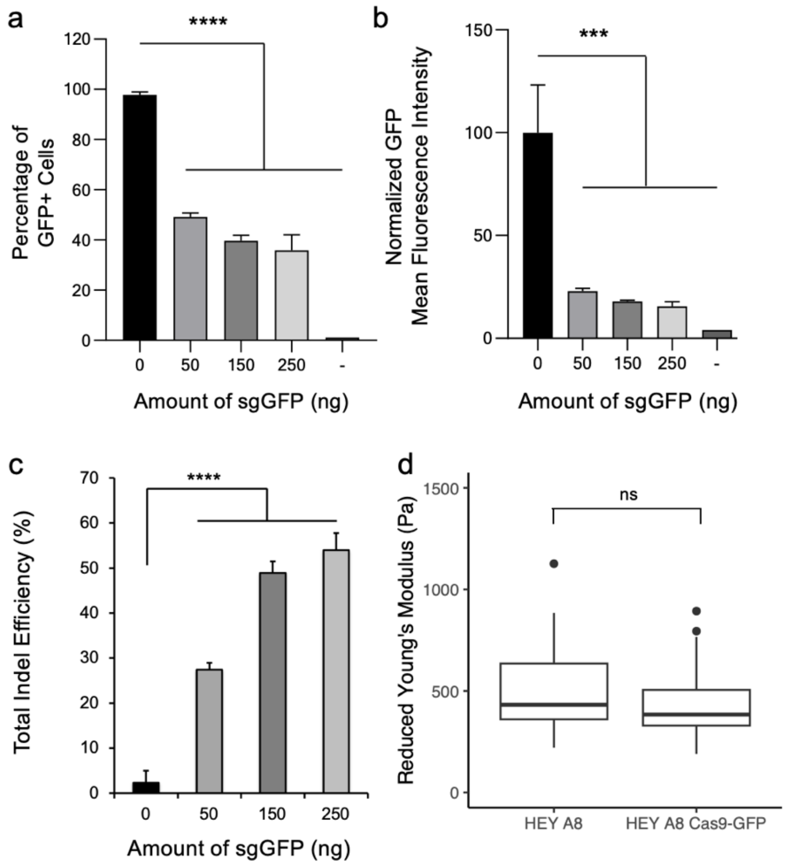

154

bioRxiv preprint doi: https://doi.org/10.64898/2026.02.12.705447; this version posted February 12, 2026. The copyright holder for this preprint (which was not certified by peer review) is the author/funder, who has granted bioRxiv a license to display the preprint in perpetuity. It is made available under aCC-BY 4.0 International license.

Supplemental Figure 1 – Cas9-expressing cells capable of gene editing – a) The addition of sgGFP to Cas9 and GFP expressing HEY A8 cells results in the significant reduction GFP+ cells as measured by flow cytometry (Tukey HSD post-hoc test, ****, $p < 0.0001$). "–" indicates cells from the HEY A8 cell line that do not express Cas9 and GFP. b) The addition of sgGFP to HEY A8-Cas9-GFP cells also results in significant reduction of mean fluorescent intensity (MFI) of each sample implying successful knockout of GFP region (Tukey HSD post-hoc test, ***, $p < 0.001$). "–" indicates cells from the HEY A8 cell line that do not express Cas9 and GFP. c) TIDE analysis to determine the insertion-deletion efficiency showed significant increase after the addition of varying amounts of sgGFP to HEY A8-Cas9-GFP cells (Tukey HSD post-hoc test, ****, $p < 0.0001$). d) Atomic force microscopy shows introduction of the Cas9-GFP cassette did not affect the reduced Young's modulus of the cell line. ($n=40$ per cell line, Welch two-sample t-test, $p > 0.05$.)

bioRxiv preprint doi: https://doi.org/10.64898/2026.02.12.705447; this version posted February 12, 2026. The copyright holder for this preprint (which was not certified by peer review) is the author/funder, who has granted bioRxiv a license to display the preprint in perpetuity. It is made available under aCC-BY 4.0 International license.

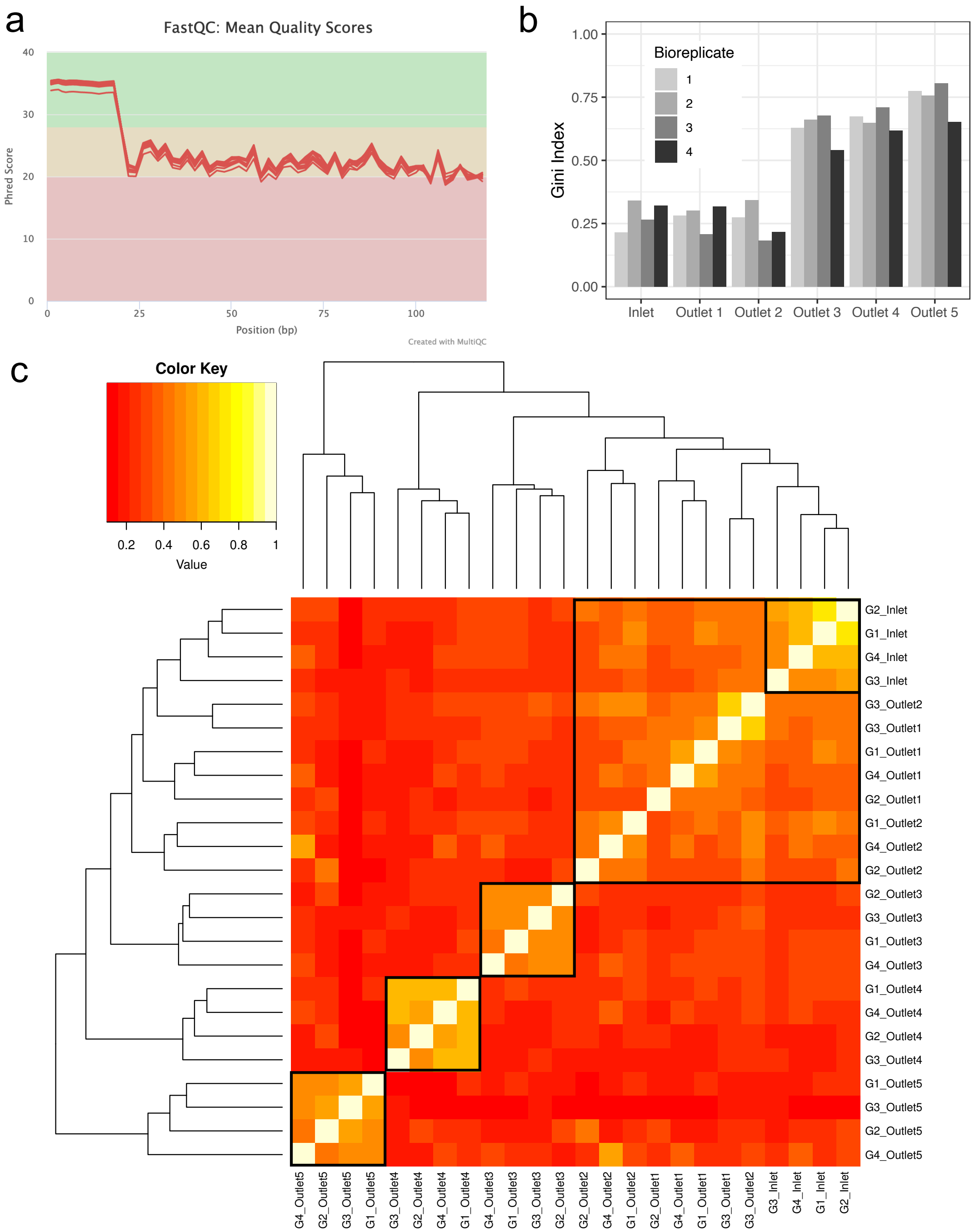

Supplemental Figure 2 – Quality control for DNA sequencing demonstrates successful amplification of sgRNA regions and positive selection for Outlets 3, 4, and 5 – a) FastQC results show mean read quality Phred scores indicating high quality reads for first 20 base pairs corresponding with the 20 bp long variable sgRNA region. b) Barplot of Gini index shows increase in positive selection for Outlets 3, 4, and 5. A low Gini index indicates more even sgRNA distribution whereas a high Gini index indicates higher selection of unique sgRNAs. c) Heatmap and hierarchal clustering of samples shows clustering of biological replicates as well as distinct nature of Outlets 3, 4, and 5 compared to similarity between Outlets 1, 2, and Inlet.

bioRxiv preprint doi: https://doi.org/10.64898/2026.02.12.705447; this version posted February 12, 2026. The copyright holder for this preprint (which was not certified by peer review) is the author/funder, who has granted bioRxiv a license to display the preprint in perpetuity. It is made available under aCC-BY 4.0 International license.

a

Functional Category	Sorting Terms Found in GO Annotations
Cytoskeleton	”cytoskeleton”, “microtubule”, “actin”, “spindle”, “filament”, “shape”, “actomyosin”, “cilium”, “tubulin”
Cell Cycle	“cell cycle”, ”mitotic”, “mitosis”, “meiosis”, “chromosome segregation”, “checkpoint”, “chromatid”, “cell division”
DNA_RNA_Metabolism	"DNA", "RNA", "transcription", "replication", "repair", "splicing", "nucleotide", "chromosome", "chromatin"
SpecificSignaling	"MAPK", "Wnt", "Notch", "PI3K", "JNK", "TGFb", "phosphatidylinositol 3-kinase"
Signaling	"signal", "kinase", "phosphorylation", "receptor", "cascade"
Transcription_Translation	"translation", "ribosome", "ribosomal", "protein synthesis", "transcription factor", "RNA polymerase", "gene expression"
Immune	"immune", "cytokine", "interferon", "antigen", "T cell", "B cell", "inflammatory", "MHC", "immunity", "natural killer", "complement"
Metabolism	"metabolic process", "biosynthetic", "catabolic", "oxidation", "glycolysis", "lipid", "amino acid","mitochondri", "autophagy", "glycolytic"
Transport	transport", "trafficking", "vesicle", "endocytosis", "exocytosis", "Golgi", "localization", "import", "export"
Adhesion_Migration	"adhesion", "migration", "motility", "junction", "extracellular matrix", "integrin", "epithelial to mesenchymal transition", "chemotaxis", "collagen", "matrix"
Stress_Response	"stress", "response", "damage", "unfolded protein", "oxidative", "heat shock"
Development	"differentiation", "development", "morphogenesis", "fate", ”commitment", "ogenesis”
Apoptosis	"apoptosis", "apoptotic", "senescence"

b

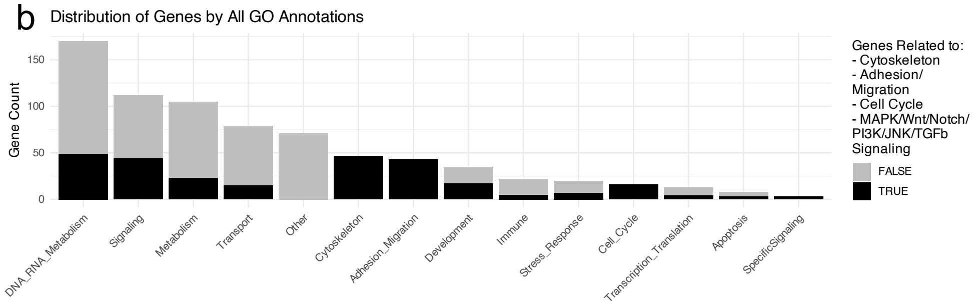

c

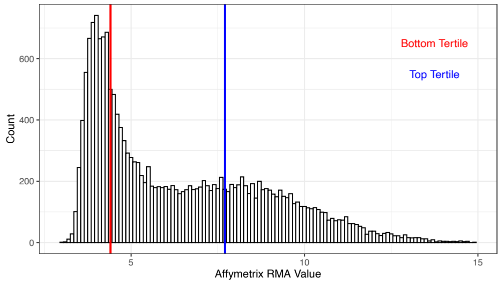

Supplemental Figure 3 – GO annotation mining and population expression shows relevant functional groups and highly expressed genes – a) Table defining functional categories based on presence of sorting terms in GO annotations for biological processes, cellular components, and molecular functions. GO annotations without any of these terms were defined as “Other”. b) Distribution of genes by GO annotations. Main category was defined as the most frequent category for all GO annotations (or second most frequent if the main category was “Other”). Additionally, if any annotations related to the categories of Cytoskeleton, Adhesion & Migration, Cell Cycle, or Specific Signaling were associated with a gene, those genes were flagged. c) Histogram showing genes by expression value in HEY A8 cell line using Affymetrix Gene Chip data from the Gene Expression Omnibus (GSM887080).

bioRxiv preprint doi: https://doi.org/10.64898/2026.02.12.705447; this version posted February 12, 2026. The copyright holder for this preprint (which was not certified by peer review) is the author/funder, who has granted bioRxiv a license to display the preprint in perpetuity. It is made available under aCC-BY 4.0 International license.

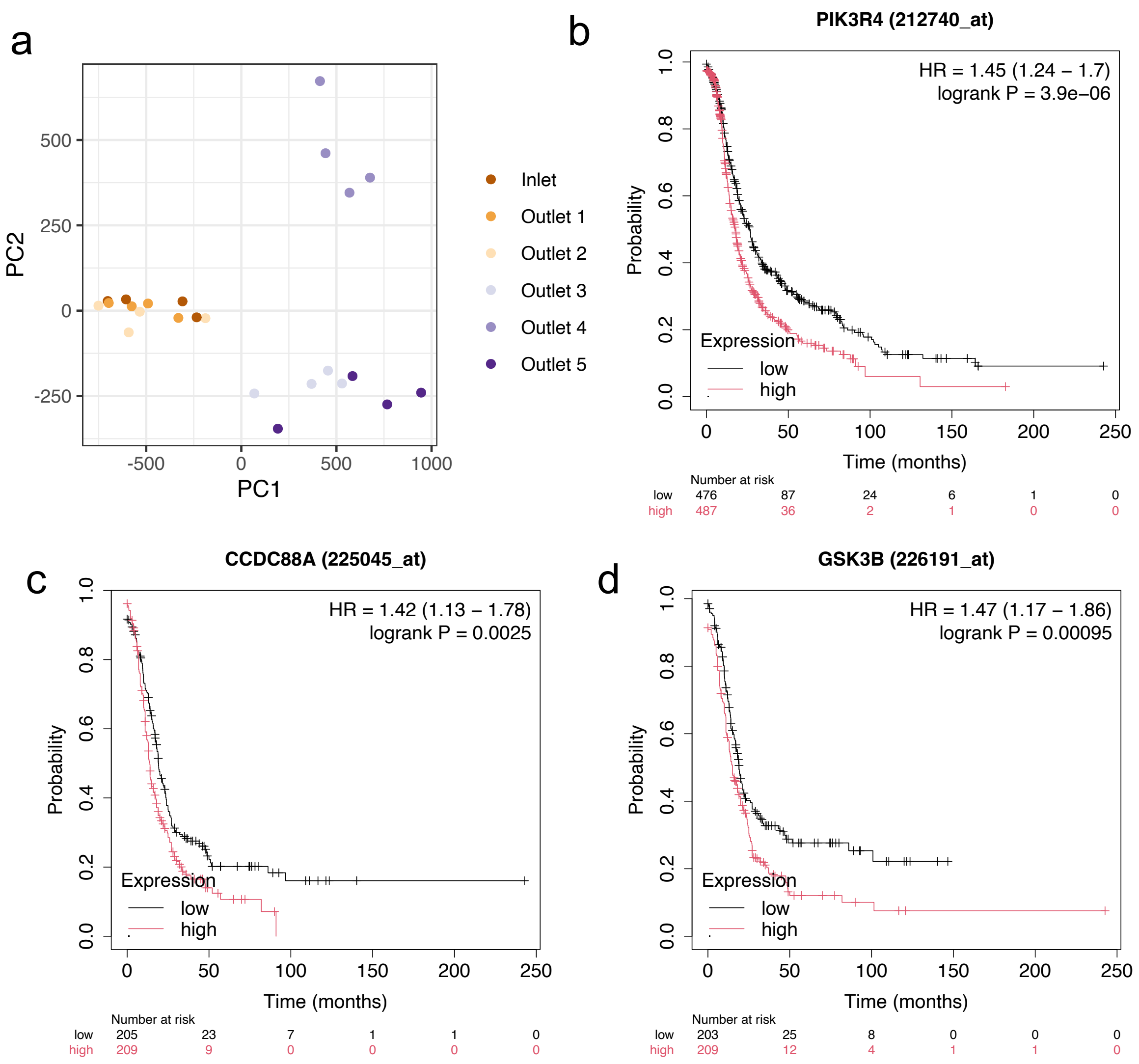

Supplemental Figure 4 – Determining genes of interest for validation – a) Principal component analysis showing clustering of inlet, outlet 1, and outlet 2 samples, clustering of outlet 3 and outlet 5 samples, and distinct clustering of outlet 4 samples. This result motivated the selection of genes enriched in outlet 4 for follow up confirmational experiments. Kaplan- Meier progression free survival curves for b) *PIK3R4,* c) *CCDC88A*, and d) *GSK3B* expression in ovarian cancer patients.
